# Understanding Cognition of Indian Superagers Through Handgrip Strength Examination

**DOI:** 10.1002/alz.091271

**Published:** 2025-01-09

**Authors:** Dwaiti Roy, Monisha S, Thomas Gregor Issac

**Affiliations:** ^1^ Centre for Brain Research, Indian Institute of Science, Bengaluru, Karnataka India; ^2^ Centre for Brain Research, Indian Institute of Science, Bangalore, Karnataka India

## Abstract

**Background:**

Physiological and mental functions decline as the person ages. However, studies have found that a group of people who are more than 80 years have better cognitive functioning than their peers and also similar to 2‐3 decades younger counterpart group. These people are termed as “Superagers”. Earlier research has revealed that handgrip strength can predict frailty and eventually decline in cognition in healthy individuals. It is important to determine the patterns of functioning and risk factors of these superagers to ensure a greater quality of life for them in the future. Therefore, this study aims to look for the patterns of handgrip strength among the superagers in comparison to the typical agers.

**Method:**

The analysis was done using baseline data from Tata Longitudinal Study of Aging (CBR‐TLSA) cohort. From this cohort, 65 octogenarians were identified. The cognition was assessed through Addenbrooke’s Cognitive Examination (ACE III) and Trail Making Test‐B(TMT‐B) The superagers were classified from these octogenarians based on Northwestern Criteria: having equal to or greater score in memory test than the 50‐65 gender and education matched age group and scores of tests of other cognitive domains not less than 1 SD compared to the same age group. Handgrip strength was measured by Camry EH101 hand dynamometer.

**Results:**

Among 65 octogenarians 14 were identified as superagers from the TLSA cohort based on the Northwestern Criteria. The educational level was higher in superager group (p=0.022). They also exhibited higher handgrip strength in both right and left hand in trial 1 (p=0.001, p=0.003 respectively) as well as in trial 2 (p=0.001, p=0.001 respectively).

**Conclusion:**

The results indicate a higher level of handgrip strength in superagers in both right and left hand. This indicates that there is less chance of frailty in superagers which in turn points to a lower risk of developing cognitive deficit.

